# Identification of 15 novel risk loci for coronary artery disease and genetic risk of recurrent events, atrial fibrillation and heart failure

**DOI:** 10.1038/s41598-017-03062-8

**Published:** 2017-06-05

**Authors:** Niek Verweij, Ruben N. Eppinga, Yanick Hagemeijer, Pim van der Harst

**Affiliations:** 1 University of Groningen, University Medical Center Groningen, Department of Cardiology, 9700 RB Groningen, The Netherlands; 2 University of Groningen, University Medical Center Groningen, Department of Genetics, 9700 RB Groningen, The Netherlands; 3 Durrer Center for Cardiogenetic Research, Netherlands Heart Institute, 3511GC Utrecht, The Netherlands

## Abstract

Coronary artery disease (CAD) is the major cause of morbidity and mortality in the world. Identification of novel genetic determinants may provide new opportunities for developing innovative strategies to predict, prevent and treat CAD. Therefore, we meta-analyzed independent genetic variants passing P <× 10^−5^ in CARDIoGRAMplusC4D with novel data made available by UK Biobank. Of the 161 genetic variants studied, 71 reached genome wide significance (p < 5 × 10^−8^) including 15 novel loci. These novel loci include multiple genes that are involved in angiogenesis (*TGFB1, ITGB5, CDH13* and *RHOA*) and 2 independent variants in the *TGFB1* locus. We also identified *SGEF* as a candidate gene in one of the novel CAD loci. SGEF was previously suggested as a therapeutic target based on mouse studies. The genetic risk score of CAD predicted recurrent CAD events and cardiovascular mortality. We also identified significant genetic correlations between CAD and other cardiovascular conditions, including heart failure and atrial fibrillation. In conclusion, we substantially increased the number of loci convincingly associated with CAD and provide additional biological and clinical insights.

## Introduction

Coronary artery disease (CAD) is a major burden of morbidity and mortality to Western society^1^. CAD is driven by a complex interplay of multiple genetic and environmental factors that jointly give rise to a plethora of molecular interactions resulting in a complex and heterogeneous phenotype. The hallmark of CAD is the development and progression of atheromatous narrowing of the coronary artery with an increasing risk of plaque rupture, resulting in acute coronary occlusion. Current preventive therapy for individuals at risk is directed towards the management of their lipid profile, blood pressure and promoting a healthy lifestyle. Genome-wide association studies (GWAS) have rapidly expanded our knowledge and provided novel leads to gain insights into human biology, optimize risk management and devise new therapeutic strategies^2^. To date, 57 loci have been reported by genome-wide association studies for CAD, mainly driven by efforts of the CARDIoGRAM- and C4D-consortia^3^. These genetic associations have identified genes that are among the targets of known and possible novel CAD therapies such as *LDLR* and *HMGCR* (HMG-coA reducatase inhibitors, statins), *PCSK9* (PCSK9 inhibitors) and *IL6R* (Tocilizumab)^4, 5^. Genetic association analyses have also identified therapeutic targets for many other conditions as well (reviewed by Plenge *et al*.^4^).

To further build upon our biological knowledge of CAD, to facilitate the identification of additional therapeutic targets, and to gain novel insights in the causal relationships between other cardiovascular phenotypes, continuous efforts directed at expanding the number of genetic regions associated with CAD are of paramount importance. Therefore, we set 3 goals. 1) Validate and identify novel loci by follow-up of the top-signals identified in the previous GWAS by the CARDIoGRAM-C4D consortium 2) determine biological pathways and candidate genes underlying the genome wide associated loci and 3) evaluate the association of the variants with common risk factors of CAD and common cardiovascular disorders to gain more insight into potential mediators of CAD per locus and trait.

## Results

First, we identified UK Biobank individuals with and without CAD. The prevalence and incidence of CAD conditions and events was captured by data collected at the Assessment Centre in-patient Health Episode Statistics (HES) and at any of the visits. A detailed definition of CAD can be found in the methods section and supplementary material.

Naturally, non-CAD individuals defined the control population but to improve statistical power we excluded individuals from the control population if their mother, father or sibling was reported to suffer from ‘heart disease′. We validated this approach by constructing a genetic risk score based on the 57 previously reported loci weighted with the effect estimates of the CARDIoGRAMplusC4D 1000 Genomes analysis assuming an additive model. The genetic risk score was associated with a family history of heart disease (n_cases_ = 71,263, n_controls_ = 76,535, p = 3 × 10^−128^) in UK Biobank. Moreover, increased significance was observed for the association between the genetic risk score and CAD after excluding participants in the control group based on a family history (p = 5 × 10^−183^), compared to including these individuals (p = 2 × 10^−147^). Indicating that incorporating family history into the phenotype definition increases statistical power to detect associations between genetic variants and CAD.

This approach identified a total of 10,898 CAD cases and 76,535 non-CAD controls in UK Biobank that were imputed to the 1000 Genomes and UK10K reference panel^6^. The average age for CAD identified participants was 61.5 years and 55.8 for the controls. Detailed baseline characteristics are presented in Supplementary Table 1. To account for potential population stratification and genotyping differences, all associations in this manuscript were adjusted for the first 15 principle components, genotyping chip, gender and age.

### Replication and identification of novel CAD loci

**Figure 1:**
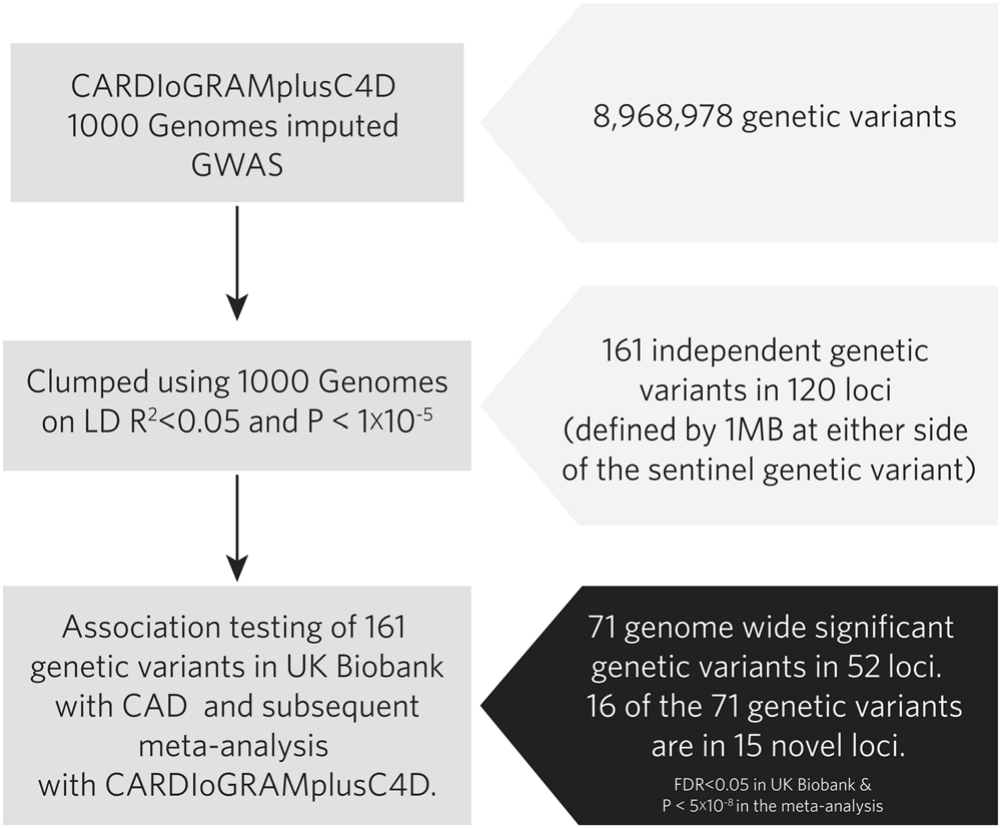
Flowchart of the study design.

To date, 57 loci have been associated with CAD^3^. We performed logistic regressions between CAD status and these 57 previously reported CAD loci: 42 loci replicated at FDR < 0.05 in UK Biobank (Supplementary Table 2). A schematic overview of the 2-stage design to identify new CAD loci is presented in Fig. 1. We first clumped genetic variants on LD (r^2^ < 0.05, 1000 Genomes phase 1 v3 European panel) that reached a P value of < 1 × 10^−5^ in the latest CARDIoGRAMplusC4D GWAS. This resulted in 161 independent sets of variants sharing 120 independent loci (defined as 1 MB at either side of the sentinel genetic variant; Supplementary Table 3). Seventy-one genetic variants in 52 loci were significantly associated (FDR < 0.05) with CAD in UK Biobank, were directionally concordant with CARDIoGRAMplusC4D, and were genome wide significant (p < 5 × 10^−8^) in the joint (meta-) analysis of UK Biobank and CARDIoGRAMplusC4D. Of these 52 loci, 37 were previously reported as genome-wide significant loci for CAD leaving 15 novel genome wide significant loci (Table 1, Supplementary Figure 1). All of the 15 novel loci were common. The minor allele frequency was above 7.6% with relatively weak effect sizes.

**Table 1:** Fifteen novel genome wide associated loci for coronary artery disease. Data presented here is from the meta-analysis; full summary statistics are available in supplementary Table 3.

Region	Genetic variant	EA/NEA	EAF	OR (95% CI)	P Value	Gene
1q21.3	rs11810571	G/C	0.787	1.069 (1.05-1.09)	1.72 × 10^−10^	TDRKH^n,e^
3p21.31	rs7623687	A/C	0.859	1.069 (1.04-1.09)	3.28 × 10^−08^	RHOA^n^, AMT^n^, TCTA^n^, CDHR4^c^, KLHDC8B^d^
3q21.2	rs142695226	G/T	0.136	1.078 (1.05-1.10)	1.70 × 10^−10^	UMPS^n,e^, ITGB5^n,d^
3q25.2	rs433903	G/A	0.857	1.081 (1.06-1.11)	6.06 × 10^−10^	SGEF(Arhgef26)^n^, DHX36^e^
4q21.21	rs10857147	T/A	0.269	1.061 (1.04-1.08)	4.29 × 10^−10^	PRDM8^n^, FGF5^n^
4q27	rs11723436	G/A	0.305	1.053 (1.04-1.07)	7.01 × 10^−09^	MAD2L1^n^, PDE5A^e^
4q31.21	rs35879803	C/A	0.702	1.051 (1.03-1.07)	3.83 × 10^−08^	ZNF827^n,e^
6p22.3	rs35541991	C/CA	0.312	1.049 (1.03-1.07)	2.57 × 10^−08^	HDGFL1^n^
11p15.2	rs1351525	T/A	0.674	1.049 (1.03-1.07)	4.09 × 10^−08^	ARNTL^n,c,e^
12q13.13	rs11170820	G/C	0.076	1.098 (1.06-1.13)	4.09 × 10^−08^	HOXC4^n^
12q24.31	rs2244608	G/A	0.349	1.056 (1.04-1.07)	1.86 × 10^−10^	HNF1A^nc^, OASL^d^
14q24.3	rs3832966	I/D	0.458	1.054 (1.04-1.07)	5.80 × 10^−10^	TMED10^n,e^, ZC2HC1C^e^, RPS6KL1^e^, NEK9^e^, EIF2B2^e^, ACYP1^e^
16q23.1	rs33928862	D/I	0.506	1.049 (1.03-1.07)	2.47 × 10^−08^	BCAR1^n,e,d^
16q23.3	rs7500448	A/G	0.772	1.069 (1.05-1.09)	4.83 × 10^−11^	CDH13^n,e,d^
19q13.2	rs138120077	D/I	0.140	1.072 (1.05-1.10)	9.44 × 10^−09^	HNRNPUL1^n,e^, TGFB1^e,d^, CCDC97^e^
19q13.2	rs8108632*	T/A	0.484	1.052 (1.03-1.07)	9.54 × 10^−09^	TGFB1^n,d^, B9D2^n^

Abbreviations: EA = effect allele, NEA = Non-effect allele,*, EAF* = *effect allele frequency*, OR = Odds Ratio*, CI* = *confidence interval*, *I* = *Indel, D* = *Deletion. Candidate gene superscripts indicate the method of identification (n* = nearest gene, c = coding gene, d = depict gene, e = eQTL gene). *Denotes the secondary signal in locus of region 19q13.2.

### Candidate genes and pathway analyses

**Figure 2:**
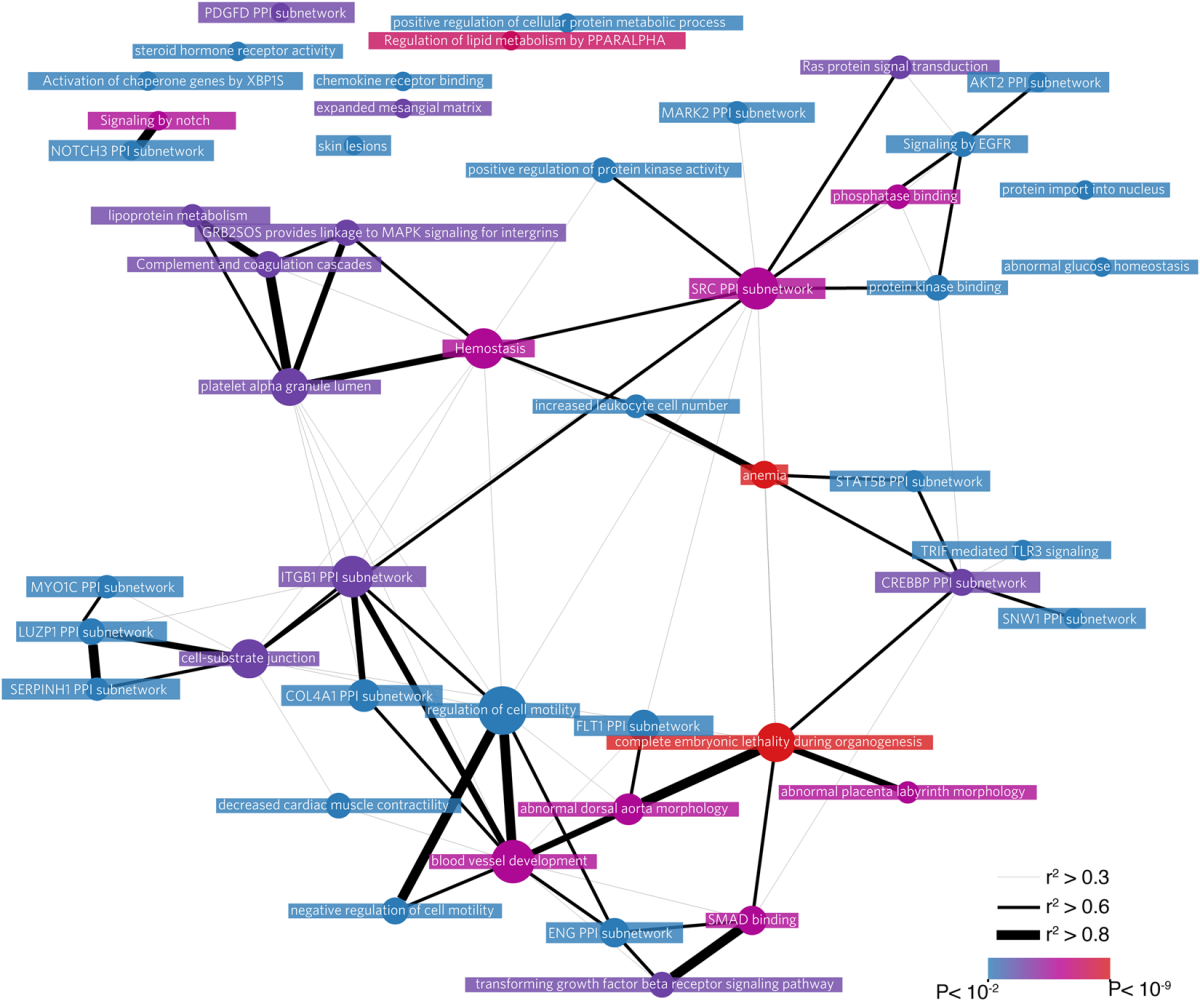
Gene-networks of the meta gene-sets that DEPICT prioritized at FDR < 0.05. Sizes of the nodes reflect the eigenvector centrality, an indicator of a node’s centrality in the network.

We prioritized 104 candidate genes in the 52 loci: 70 genes were prioritized based on proximity (the nearest gene and any additional gene within 10 kb), 13 genes by coding genetic variants in linkage disequilibrium (*r* ^2^ > 0.8) with the sentinel genetic variant (Supplementary Table 4) and 51 genes based on expression quantitative trait loci (eQTL) analyses (Supplementary Table 5). Finally, 25 candidate genes were prioritized based on DEPICT analyses^7^ (Supplementary Table 6). The DEPICT framework also identified 458 reconstituted gene sets that can be captured in 48 meta-genesets (Fig. 2), the most significant gene set was ‘abnormal vitelline vasculature morphology’ (Supplementary Table 7 **)**; ‘Arteries’ was the only significantly prioritized tissue-type (FDR < 0.01, Supplementary Table 8). Network analysis identified ‘regulation of cell motility’ as the meta-geneset that was most central among all meta-genesets, together with ‘negative regulation of cell motility’ and ‘blood vessel development’, suggesting these pathways play an important role in CAD. More specific processes identified by DEPICT included hemostasis, anemia and increased leukocyte cell number. The function of each novel candidate gene has been summarized in the Supplementary Note.

### Mediating effects of CAD variants

CAD is a complex multifactorial disease, sharing biology with other atherosclerotic manifestations and vascular diseases. Therefore, we examined the association between genetic risk for CAD and several common cardiovascular phenotypes. We constructed a weighted genetic risk score by summing the number of CAD increasing risk alleles after multiplying the alleles with the corresponding β (based on the CARDIoGRAMplusC4D GWAS^3^). The genetic risk score that was based on the 71 independent genetic variants was associated with multiple other cardiovascular phenotypes and risk factors in UK Biobank (Table 2). At baseline, the genetic risk score was significantly associated with BMI, height, systolic blood pressure, mean arterial pressure, pulse pressure and heart rate. The genetic risk score was also associated with the presence of heart failure, hypertension, smoking, device implantation, cerebral infarction (including transient ischemic attack), atrial fibrillation and diabetes; whereas cardiomyopathy, diastolic blood pressure and arterial stiffness index were not significantly associated. We also tested whether the genetic risk score could predict cardiovascular mortality, death from coronary artery disease, death from myocardial infarction, and death from heart failure. The genetic risk score significantly predicted these outcomes in cox’s proportional hazards models (Table 3). In addition, the genetic risk score predicted recurrent CAD events (n = 3,733) in participants with a history of CAD (n = 6,440; HR = 1.10, Confidence interval 1.03–1.19, p = 0.009). Results were the same for a genetic risk score based on 57 previously known loci (Supplementary Table 9).

**Table 2:** Associations in UK Biobank (N = 143,936) between the genetic risk score based on the 71 genome wide significant CAD variants and cardiovascular profile.

Phenotype	N individuals (%)	Beta (linear Regression) or odds ratio (logistic regression) (95% CI)	P value
Body-mass index	143,442 (99.7%)	−0.08(−0.14 to −0.02)	5.80 × 10^−03^
Height	143,595 (99.8%)	−0.16(−0.24 to −0.09)	2.78 × 10^−05^
Resting heart rate	135,946 (94.4%)	−0.45(−0.59 to −0.31)	2.03 × 10^−10^
Blood pressure			
Systolic	143,770 (99.9%)	0.61(0.40 to 0.82)	1.27 × 10^−08^
Diastolic	143,770 (99.9%)	−0.02(−0.14 to 0.10)	7.60 × 10^−01^
Pulse pressure	143,770 (99.9%)	0.63(0.48 to 0.78)	1.65 × 10^−16^
Mean arterial pressure	143,770 (99.9%)	0.19(0.05 to 0.33)	7.14 × 10^−03^
Arterial stiffness index	54,184 (37.6%)	−0.02(−0.08 to 0.04)	5.38 × 10^−01^
Smoking current	18,282 (14.5%)	−0.05(0.91 to 0.98)	5.45 × 10^−03^
Medical Conditions			
Coronary Artery Disease	10,898 (8.2%)	2.21(2.11 to 2.32)	1.76 × 10^−237^
Hypertension	48,927 (51.5%)	1.18(1.15 to 1.22)	5.89 × 10^−35^
Diabetes	10,486 (7.9%)	1.10(1.05 to 1.16)	5.91 × 10^−05^
Myocardial Infarction	5,145 (3.7%)	2.35(2.20 to 2.51)	2.05 × 10^−143^
Heart failure	2,143 (1.5%)	1.43(1.29 to 1.58)	2.76 × 10^−12^
Cardiomyopathy	522 (0.4%)	1.02(0.84 to 1.25)	8.39 × 10^−01^
Atrial fibrillation/flutter	5,279 (3.8%)	1.10(1.03 to 1.18)	2.90 × 10^−03^
Cerebral Infarction and TIA	4,043 (2.9%)	1.16(1.08 to 1.25)	4.68 × 10^−05^
Device implantation	1,606 (1.1%)	1.51(1.35 to 1.69)	1.76 × 10^−12^
Medication			
Beta-blockers	10,576 (7.9%)	1.42(1.36 to 1.49)	1.15 × 10^−49^
Calcium channel-blockers	10,993 (8.3%)	1.17(1.12 to 1.23)	3.19 × 10^−11^

Effect estimates with 95% Confidence Interval (CI) are shown as odds ratios for categorical variables (current smoking, cardiovascular disease, atherosclerosis, hypercholesterolemia, hypertension, diabetes, myocardial infarction, heart failure, atrial fibrillation/flutter, cerebral Infarction and TIA, device implantation, beta-blockers and calcium-channel blockers) and β estimates for quantitative variables (body-mass index, resting heart rate, systolic and diastolic blood pressure, pulse pressure, mean arterial pressure and arterial stiffness index). Abbreviation: N = Number, CI = Confidence Interval, TIA = Transient Ischemic Attack.

**Table 3:** Cox survival model predicting hazard of death using the genetic risk score based on the 71 genome wide significant CAD variants.

Phenotype	N deaths (%)	Hazard Ratio (95% CI)	P value
Coronary Artery Disease	723 (0.5%)	1.75 (1.48 to 2.08)	4.90 × 10^−11^
Myocardial Infarction	210 (0.1%)	1.93 (1.41 to 2.63)	3.40 × 10^−05^
Heart failure	219 (0.2%)	1.39 (1.02 to 1.88)	3.69 × 10^−02^
Cardiomyopathy	40 (0.0%)	1.18 (0.58 to 2.40)	6.48 × 10^−01^
Cerebral Infarction and TIA	124 (0.1%)	1.32 (0.87 to 1.98)	1.89 × 10^−01^
All cause mortality	4373 (3.0%)	1.02 (0.95 to 1.09)	5.65 × 10^−01^
Cardiovascular mortality (as primary cause of death)	892 (0.6%)	1.46 (1.26 to 1.70)	9.08 × 10^−07^

Abbreviations: N = Number, CI = Confidence Interval, TIA = Transient Ischemic Attack.

BOLT-REML^8^ was used to assess the cumulative contribution of common genetic variants to CAD risk, and to estimate the degree of genetic correlation between CAD and other cardiovascular phenotypes in UK Biobank. We estimated the heritability of CAD by genome wide genetic variants, *h* ^*2*^_*g*_, to be 0.063 (SE = 0.0046), which is 0.22 on the liability scale (with a prevalence of 0.076, based on UK Biobank). CAD and almost all other studied cardiovascular phenotypes were genetically correlated, which led to comparable conclusions as our genetic risk score analyses. Narrow sense heritability estimates of all studied cardiovascular phenotypes and estimates of shared genetic correlations with CAD are available in Table 4.

**Table 4:** Heritability estimates for cardiovascular traits and the shared heritability between each trait and CAD in all UK Biobank Participants based on common genetic variation under the additive model (*h* ^*2*^_*g*_). For dichotomous traits, the heritability on the observed 0-1 scale was transformed to *h* ^*2*^_*g*_ on the unobserved continuous liability scale by a linear transformation.

Phenotype	*h*2_g_(Se)	P-*h*2_g_	Genetic correlation with CAD (Se)	P-correlation
Body-mass index	0.316 (0.005)	0	0.289 (0.026)	3.67 × 10^−28^
Height	0.614 (0.004)	0	−0.142 (0.019)	5.93 × 10^−13^
Resting heart rate	0.204 (0.005)	0	0.099 (0.033)	4.64 × 10^−03^
Blood pressure				
Systolic	0.198 (0.005)	0	0.380 (0.034)	8.04 × 10^−29^
Diastolic	0.197 (0.005)	0	0.316 (0.034)	3.15 × 10^−20^
Pulse pressure	0.220 (0.005)	0	0.265 (0.030)	8.08 × 10^−18^
Mean arterial pressure	0.191 (0.005)	0	0.380 (0.035)	3.70 × 10^−27^
Arterial stiffness index	0.082 (0.012)	2.13 × 10^−11^	0.075 (0.118)	3.26 × 10^−01^
Smoking current	0.238 (0.012)	3.06 × 10^−87^	0.258 (0.043)	8.30 × 10^−09^
Medical Conditions				
Coronary Artery Disease	0.216 (0.016)	8.77 × 10^−42^	—	—
Hypertension	0.310 (0.008)	0	0.577 (0.031)	1.39 × 10^−75^
Diabetes	0.345 (0.017)	2.90 × 10^−95^	0.412 (0.041)	2.88 × 10^−23^
Myocardial Infarction	0.190 (0.025)	4.21 × 10^−14^	1.000 (0.035)	2.40 × 10^−174^
Heart failure	0.098 (0.044)	3.49 × 10^−02^	0.679 (0.158)	4.04 × 10^−05^
Cardiomyopathy	0.071 (0.134)	3.47 × 10^−01^	0.462 (0.483)	2.53 × 10^−01^
Atrial fibrillation/flutter	0.238 (0.025)	1.92 × 10^−21^	0.323 (0.058)	8.29 × 10^−08^
Cerebral Infarction and TIA	0.090 (0.028)	2.62 × 10^−03^	0.635 (0.134)	4.93 × 10^−06^
Device implantation	0.074 (0.055)	1.61 × 10^−01^	0.678 (0.261)	1.36 × 10^−02^
Medication				
Beta-blockers	0.156 (0.016)	1.94 × 10^−22^	0.818 (0.053)	6.26 × 10^−53^
Calcium channel-blockers	0.251 (0.016)	6.41 × 10^−55^	0.547 (0.049)	2.76 × 10^−28^

Abbreviations: *h*2_g_ = heritability based on genome wide variation, Se = Standard error, TIA = Transient Ischemic Attack

(h2g); standard error (SE); N.A. ; not applicable.

To gain further insights into potential mediating mechanisms at the genetic variant level, we queried the GWAS-catalog for previously reported genome wide associations: for the novel loci, genetic variants in linkage disequilibrium (r^2^ > 0.5) with rs10857147 (*FGF5*) was previously associated with blood pressure and serum urate levels; rs2244608 (*HNF1A*/*OASL*) with a wide range of biomarkers including lipids and urate levels; rs3832966 (*TMED10*/*NEK9*) with adult stature; rs1351525 with menarche; rs33928862 with pulmonary function; and rs8108632 (*B9D2*/*TGFB1*/*AXL*) with migraine and colorectal cancer risk (Supplementary Table 10). Furthermore, we performed association testing in the CAD controls of UK Biobank (N = 76,535) to reduce potential reverse causation of CAD for the following traits: systolic- and diastolic blood pressure, mean arterial pressure, pulse pressure, arterial stiffness index, heart rate, smoking and diabetes. We also performed lookups in previously published datasets of large GWAS: lipids^9^, BMI^10^, Hip circumference^11^, waist-hip ratio^11^ (adjusted for BMI), results are presented in Supplementary Table 11. Of the 71 genetic variants, 63 were nominally associated (p < 0.05) with one or more phenotypes. These lookups confirmed our findings of the GWAS-catalog query; rs2244608 is highly associated with total cholesterol and LDL (p = 9 × 10^−21^), rs10857147 (*FGF5*) is associated with blood pressure (p = 2 × 10^−13^) but also identified novel associations for the novel loci such as rs7500448 (pulse pressure, p = 4 × 10^−11^), rs1351525 (Systolic blood pressure p = 7 × 10^−7^) and rs33928862 (Systolic blood pressure p = 5 × 10^−4^). It also highlighted 21 of 71 variants without any association (P > 0.05) with blood pressure or lipid traits (Supplementary Table 11).

## Discussion

Using a 2-stage design, adding 10,898 new cases and 76,535 controls to the 60,801/130,681 controls/cases previously studied by the CARDIoGRAMplusC4D consortium, we identified 15 novel loci reaching genome wide significance^3^. The variants of these 15 loci were common, with generally low effect sizes. In keeping with previous observations, our strategy did not reveal CAD variants of low frequency (MAF < 1-0.05%), suggesting that even other reference sets, techniques or larger sample sizes are required^3^. We added a relatively modest increase in cases (17.9%) compared to CARDIoGRAMplusC4D data but the number of additional controls was substantially higher (58.6%) and by filtering on a family history of ‘heart disease’ we might have decreased the number of misclassifications. These aspects of our strategy may have contributed to the relative large number of novel CAD loci compared to the latest CARDIoGRAMplusC4D that identified 10 new loci. Within UK Biobank we observed that the genetic risk score significantly predicts - and has a shared heritability with - a range of cardiovascular phenotypes, illustrating for example that genetically predicted CAD also increases risk for heart failure and atrial fibrillation, in line with observations from clinical practice.

Of the novel prioritized candidate genes, some have been previously reported for their involvement in blood vessel development. For example, *RHOA*, part of the Ras protein family, is involved in a multitude of cellular processes via the Rho-kinase pathway which has a primary role in the regulation of contraction in vascular smooth muscle cells and promoting development of vascular remodeling^12^. *CDH13* which encodes T-cadherin, is a regulator of vascular wall remodeling, angiogenesis and is essential for adiponectin’s vascular actions^13^. *TGFB1*, one of the most widely studied genes, is crucial in embryonic development and tissue homeostasis. The role of *TGFB1* in angiogenesis is a fact and long thought to play a role in CAD development, but the exact molecular pathways are hard to tackle due to the complex and multifactorial nature^14^. Rs2241718 near *TGBF1* has been prioritized previously as a functional regulatory variant^15^ but is in low linkage disequilibrium (r2 < 0.05) with the two signals identified here. Identifying two independent variants in this locus provides new opportunities to study the role of *TGFB1* in CAD. The product of *ITGB5*, integrin β5 has been studied in some detail for its role in cell adhesion and integrin-mediated signaling. It is believed that *ITGB5* is able to exert pro-angiogenic effects by enhancing the binding capacity of circulating angiogenic cells to endothelial cells^16^. Our pathway analyses also suggest that factors related to angiogenesis (‘blood vessel development’ and ‘regulation of cell motility’) are indeed central among the CAD loci, supplementing previously performed pathway analyses^17^.

We also identified a novel CAD locus (rs433903) harboring *SGEF*. *SGEF* has been described to contribute to the formation of ICAM-1-induced endothelial docking structures that facilitate transendothelial migration and adhesion of leukocytes^18^. This process has an unfavorable role in atherosclerosis: *SGEF* ^-/-^ mice demonstrate a significant reduction in the formation of atherosclerotic plaque and was suggested as a novel therapeutic target, also since there appeared to be no other negative phenotypes^18, 19^. Here, we demonstrate that rs433903 near *SGEF* is associated with CAD in humans and is not convincingly associated with other phenotypes such as blood pressure and lipids. Future studies are necessary to determine the exact molecular mechanisms underlying rs433903 and whether this variant is causally implicated in CAD through mechanisms of *SGEF* to further establish *SGEF* as a new candidate target for therapy.

The majority of preventive CAD medication is currently directed towards lowering LDL cholesterol and blood pressure, both of which are also closely associated with CAD on a genetic level, and considered to be causally related^20–22^. Genetic variants lacking any association with blood pressure or lipids might be of increased interest to be considered as novel (first in class) therapeutic targets that act independently from blood pressure or lipid lowering medication. However, our analyses are limited by the associative nature. To establish further evidence of the true causal genes and mechanisms underlying each association, further functional experiments are essential.

We are the first to have observed a significant genetic correlation between CAD and heart failure. The degree of shared heritability between CAD and heart failure was estimated to be as high as 0.68. We also observed that genetic risk for CAD was strongly associated with the occurrence of heart failure due to CAD, and predicts death of heart failure with similar effects. It is well known that CAD plays a major role in heart failure, prevention of CAD is essential to maintaining functional myocyte reserve and preventing left ventricular systolic dysfunction^23^. Furthermore, a significant correlation and shared heritability was observed between the genetic risk score of CAD and increased risk of atrial fibrillation, perhaps due to atrial infarction but shared mechanisms of inflammation may also be responsible^24^.

We could not only explain death due to CAD using our genetic risk score, in line with other studies^25^, but could even predict progression of CAD as indicated by the significant association with recurrent CAD. A genetic risk score may be helpful to discriminate individuals at high risk of CAD and to direct more intensive preventive therapies. Future studies should be focused at replicating the newly identified loci and at further elucidating the molecular and pathophysiological mechanisms underlying CAD.

In summary, we report 15 novel loci, representing a 20% expansion of loci that are genome wide associated with CAD, including 2 independent variants near *TGFB1*. We also highlight widespread sharing of genetic variation between CAD and numerous other common cardiovascular diseases including atrial fibrillation and heart failure.

## Materials and Methods

### UK biobank individuals

UK Biobank recruited participants with an age range of 40–69 years that registered with a general practitioner of the UK National Health Service (NHS). Between 2006–2010, in total 503,325 individuals were included. All study participants provided informed consent and the study was approved by the North West Multi-centre Research Ethics Committee. Detailed methods used by UK Biobank have been described elsewhere.

### Ascertainment of resting coronary artery disease and controls

The prevalence and incidence of coronary artery disease conditions and events were captured by data collected at the Assessment Centre in-patient Health Episode Statistics (HES). CAD was defined using the following ICD 10 codes: I21-I25 covering ischaemic heart diseases and the following Office of Population Censuses and Surveys Classification of Interventions and Procedures, version 4 (OPCS-4) codes: K40-K46, K49, K50 and K75 which includes replacement, transluminal balloon angioplasty, and other therapeutic transluminal operations on coronary artery and percutaneous transluminal balloon angioplasty and insertion of stent into coronary artery. The exact phenotype definitions of UK Biobank are described in the supplementary note under section “Definitions used for UK Biobank analyses”. Individuals from the control group were excluded if their mother, father or sibling were reported to suffer from ‘heart disease’ to increase the true CAD/non-CAD ratio for our analysis.

### Genotyping and Imputation

Of the 500 thousand individuals with phenotype data in UK Biobank, 152,249 (25%) are currently genotyped. Genotyping, quality control and imputation was performed by UK Biobank and described in detail elsewhere^6, 26^. Briefly, genotyping of 102,326 individuals was performed using the UK Biobank Axiom array (Affymetrix), and an additional 49,923 individuals were genotyped as part of the UK Biobank Lung Exome Variant Evaluation (UK BiLEVE) project. The Welcome Trust Centre for Human Genetics performed quality control before imputation and imputed the dataset using a merged reference panel of 1000 Genomes Phase 3 and UK10K^6^. The imputed dataset consisted of 72,355,667 genetic variants. For this work, genetic variants were included only if the imputation quality was greater than 0.3 and MAF > 0.005 in line with the CARDIoGRAMplusC4D analysis, leaving 12,248,858 genetic variants. Participants were excluded based on gender mismatch, high missingness and high heterozygosity (n = 662). We also removed 8,874 individuals based on relatedness (3rd degree or closer^6^), one of each related pair was excluded based on the highest missingness.

### Statistical Analysis

We selected genetic variants for replication from the CARDIoGRAMplusC4D^3^ GWAS (downloaded from: http://www.cardiogramplusc4d.org/downloads) by filtering on p < 1 × 10^−5^ and linkage disequilibrium using the PLINK clumping procedure (*‘–clump-kb 5000 –clump-r2 0.05’*, 1000 Genomes phase 1), after which we determined the number of 2-Megabase-loci, by assigning 1 Megabase regions at either side of the highest associated variant per locus (designated the sentinel genetic variant). Logistic regression analyses between genetic variants and the 10,898 CAD cases and 76,535 controls in UK Biobank were performed after adjustments for age, sex, the first 15 Principal Components to control for population stratification, and the genotyping array used. To account for multiple testing and declare novel loci we applied a replication p of FDR < 0.05 in UK Biobank and a genome wide significance threshold of p < 5 × 10^−8^ in the inverse-variance meta-analysis between the summary statistics of UK Biobank and CARDIoGRAMplusC4D.

### Pathway analyses

The DEPICT Framework was used to identify enriched pathways, prioritize candidate genes at each loci and selects relevant tissues/cell types from co-expression networks of genes underlying the associated loci^7^ (see Pers *et al*.^7^ for a detailed description). We applied DEPICT on CARDIoGRAMplusC4D results at p < 1 × 10^–5^ which identified 194 independent loci using default settings (PLINK parameters, *‘–clump-p1 1e-5 –clump-kb 500 –clump-r2 0.01*’), containing 489 genes. The gene prioritization, gene set enrichment and tissue/cell type enrichment analyses were run using the default settings in DEPICT (1000 G dataset). We applied the affinity propagation method^27^ to identify correlated genesets and for each correlated group the exemplar-geneset, which was named ‘meta-geneset’, and used Gephi (www.gephi.org)^28^ to visualize the pearson correlation between pathways and calculate the centrality measures of each node (Fig. 2).

### Genetic risk score analyses & (shared) heritability of CAD

To study the relationship of CAD with other cardiovascular phenotypes, we created a weighted genetic risk score by summing the number of CAD risk-increasing alleles weighted (multiplied) for its β (estimated using the 1000 genomes meta-analysis^3^) of each associated genetic variant; assuming an additive effect. We performed a linear or logistic regression adjusted for age, gender, principle components and genotyping chip between the genetic risk score and cardiovascular phenotype. Cox regression analysis adjusted for age, gender, principle components and the genotyping chip was used to evaluate the predictive power of the genetic risk score on mortality and recurrent CAD events. Bivariate REML analyses were performed using BOLT-REML^8^ to estimate the heritability of CAD and the genetic correlation of CAD with other cardiovascular traits. All directly genotyped variants that passed quality control were extracted from the imputed dataset (to ensure 100% call rate) and pruned on linkage disequilibrium (*r*2 < 0.05) to obtain roughly 500 k variants, as recommended^8^. Liability scale was estimated for dichotomous traits using linear transformation^29^. Gender, age, principle components and genotyping chip we included as covariates in all analyses.

### Identification of candidate genes

We prioritized candidate genes for each of the 71-genome wide significant variants that were shared in 52 loci, based on the following criteria:

1. The nearest gene or any gene located within 10 kb of the sentinel genetic variant
2. Any gene containing protein coding variants in linkage disequilibrium (*r* ^2^ > 0.8, UK Biobank) with the sentinel genetic variant (Supplementary Table 4).
3. Expression QTL (eQTL) analyses in cis; we search for eQTLs (sentinel genetic variants or genetic variants in linkage disequilibrium, *r* ^2^ > 0.8, UK Biobank) in an eQTL dataset that was compiled from multiple tissues, including those of GTEX v6^30^, STARNET^31^ and large eQTL datasets of blood^32–34^ (see Supplementary Table 5). We only considered eQTLs for which the top-eQTL was in linkage disequilibrium (*r* ^2^ > 0.8, UK Biobank) with the sentinel genetic variant and for which the eQTL p < 1 × 10^−6^.
4. DEPICT-genes (see section “pathway analyses” for more details and Supplementary Tables 6–8).

## Electronic supplementary material

Supplementary information and figures

Supplementary tables 1–11

## References

[1] Task Force M (2013). ESC guidelines on the management of stable coronary artery disease: the Task Force on the management of stable coronary artery disease of the European Society of Cardiology. Eur. Heart J..

[2] Kullo, I. J. *et al*. Incorporating a Genetic Risk Score into Coronary Heart Disease Risk Estimates: Effect on LDL Cholesterol Levels (the MIGENES Clinical Trial). *Circulation* doi:10.1161/CIRCULATIONAHA.115.020109 (2016). 10.1161/CIRCULATIONAHA.115.020109 PMC4803581 26915630

[3] Nikpay M (2015). A comprehensive 1,000 Genomes-based genome-wide association meta-analysis of coronary artery disease. Nat. Genet..

[4] Plenge RM, Scolnick EM, Altshuler D (2013). Validating therapeutic targets through human genetics. Nat. Rev. Drug Discov..

[5] Interleukin-6 Receptor Mendelian Randomisation Analysis (IL6R MR) Consortium. The interleukin-6 receptor as a target for prevention of coronary heart disease: a mendelian randomisation analysis. *Lancet Lond. Engl*. **379**, 1214–1224 (2012). 10.1016/S0140-6736(12)60110-X PMC3316968 22421340

[6] Genotyping and quality control of UK Biobank, a large-scale, extensively phenotyped prospective resource. (2015).

[7] Pers TH (2015). Biological interpretation of genome-wide association studies using predicted gene functions. Nat. Commun..

[8] Loh P-R (2015). Efficient Bayesian mixed-model analysis increases association power in large cohorts. Nat. Genet..

[9] Global Lipids Genetics Consortium (2013). Discovery and refinement of loci associated with lipid levels. Nat. Genet..

[10] Locke AE (2015). Genetic studies of body mass index yield new insights for obesity biology. Nature.

[11] Shungin D (2015). New genetic loci link adipose and insulin biology to body fat distribution. Nature.

[12] Shimokawa H, Sunamura S, Satoh K (2016). RhoA/Rho-Kinase in the Cardiovascular System. Circ. Res..

[13] Parker-Duffen JL (2013). T-cadherin Is Essential for Adiponectin-mediated Revascularization. J. Biol. Chem..

[14] Zeng L, Dang TA, Schunkert H (2016). Genetics links between transforming growth factor β pathway and coronary disease. Atherosclerosis.

[15] Miller CL (2016). Integrative functional genomics identifies regulatory mechanisms at coronary artery disease loci. Nat. Commun..

[16] Leifheit-Nestler M (2010). Overexpression of Integrin β5 Enhances the Paracrine Properties of Circulating Angiogenic Cells via Src Kinase–Mediated Activation of STAT3. Arterioscler. Thromb. Vasc. Biol..

[17] Ghosh S (2015). Systems Genetics Analysis of GWAS reveals Novel Associations between Key Biological Processes and Coronary Artery Disease. Arterioscler. Thromb. Vasc. Biol..

[18] Samson, T. *et al*. The Guanine-Nucleotide Exchange Factor SGEF Plays a Crucial Role in the Formation of Atherosclerosis. *PLoS ONE* **8**, (2013). 10.1371/journal.pone.0055202 PMC3555862 23372835

[19] Bitoun, P. Sgef controls macular, corpus callosum and hippocampal function and development, liver homeostasis, functions of the immune system, fever response atherosclerosis and tumorogenic cell growth. (2012).

[20] Studies, T. I. C. for B. P. G.-W. A (2011). Genetic variants in novel pathways influence blood pressure and cardiovascular disease risk. Nature.

[21] Do R (2013). Common variants associated with plasma triglycerides and risk for coronary artery disease. Nat. Genet..

[22] Lieb, W. *et al*. Genetic predisposition to higher blood pressure increases coronary artery disease risk. *Hypertension* **61**, (2013). 10.1161/HYPERTENSIONAHA.111.00275 PMC3855241 23478099

[23] Doshi D (2016). Underutilization of Coronary Artery Disease Testing Among Patients Hospitalized With New-Onset Heart Failure. J. Am. Coll. Cardiol..

[24] Hu Y-F, Chen Y-J, Lin Y-J, Chen S-A (2015). Inflammation and the pathogenesis of atrial fibrillation. Nat. Rev. Cardiol..

[25] Khera, A. V. *et al*. Genetic Risk, Adherence to a Healthy Lifestyle, and Coronary Disease. *N. Engl. J. Med*. **0**, null (2016). 10.1056/NEJMoa1605086 PMC5338864 27959714

[26] Eppinga RN (2016). Identification of genomic loci associated with resting heart rate and shared genetic predictors with all-cause mortality. Nat. Genet..

[27] Bodenhofer U, Kothmeier A, Hochreiter S (2011). APCluster: an R package for affinity propagation clustering. Bioinformatics.

[28] Bastian, M., Heymann, S. & Jacomy, M. Gephi: An Open Source Software for Exploring and Manipulating Networks. (2009).

[29] Lee SH, Wray NR, Goddard ME, Visscher PM (2011). Estimating missing heritability for disease from genome-wide association studies. Am. J. Hum. Genet..

[30] Lonsdale J (2013). The Genotype-Tissue Expression (GTEx) project. Nat. Genet..

[31] Franzén O (2016). Cardiometabolic risk loci share downstream cis- and trans-gene regulation across tissues and diseases. Science.

[32] Westra H-J (2013). Systematic identification of trans eQTLs as putative drivers of known disease associations. Nat. Genet..

[33] Bonder MJ (2015). Disease variants alter transcription factor levels and methylation of their binding sites. bioRxiv.

[34] Zhernakova D (2015). Hypothesis-free identification of modulators of genetic risk factors. bioRxiv.

