# Supplementary material for: Identification of 15 novel risk loci for coronary artery disease and genetic risk of recurrent events, atrial fibrillation and heart failure
Source: Sci Rep. 2017 Jun 5;7:2761. doi: 10.1038/s41598-017-03062-8 (PMC5459820; doi:10.1038/s41598-017-03062-8)
Supplement: Supplementary file 1 — Supplementary information and figures [file 41598_2017_3062_MOESM1_ESM.pdf]

Identification of 15 novel risk loci for coronary artery disease and genetic risk of recurrent events, atrial fibrillation and heart failure

Niek Verweij[1], Ruben N. Eppinga[1], Yanick Hagemeijer[1], Pim van der Harst[1,2,3]

(1.) University of Groningen, University Medical Center Groningen, Department of Cardiology, 9700 RB Groningen, the Netherlands
(2.) University of Groningen, University Medical Center Groningen, Department of Genetics, 9700 RB Groningen, the Netherlands
(3.) Durrer Center for Cardiogenetic Research, Netherlands Heart Institute, 3511GC Utrecht, the Netherlands

Supplementary Appendix

Supplementary Note 3

Summary of the candidate genes in the novel associated loci 3

Definitions used for UK Biobank analyses 7

Supplementary Figures 10

Supplementary References 11

Supplementary Note

Summary of the candidate genes in the novel associated loci

Locus 1q21.3, rs11810571 (TDRKH): The function of TDRKH is unknown in the literature, whereas the functions of the isoforms encoded by RORC, RORγ and RORγt are complex and widely studied [1].

Locus 3p21.31, rs7623687 (RHOA, AMT, TCTA, CDHR4 and KLHDC8B): RHOA (Ras homolog gene family, member A) is a small GTPase protein from the Rho family. The effects of RHOA are not all known it is primarily associated with cytoskeleton regulation, mostly actin stress fibers formation and actomyosin contractility. The RhoA/Rho-associated coiled-coil-forming kinase (ROCK) pathway participates in acute myocardial infarction and inhibiting of this pathway with atorvastatin improves the post-infarct microenvironment[2]. The AMT gene provides instructions for making an enzyme called aminomethyltransferase. This is one of four subunits that make up glycine cleavage enzyme. This complex is active in mitochondria. Mutations in this gene are responsible for ~15% of the glycine encephalopathy[3]. Only a few studies have been studying the role of TCTA (T-cell leukemia translocation-altered), it has been reported to play a role in human tumorigenesis and osteoclastogenesis[4] and inhibit proliferation of fibroblast-like synoviocytes[5]. CDHR4 (cadherin-related family member 4) cadherins are calcium-dependent cell adhesion proteins. They preferentially interact with themselves in a homophilic manner in connecting cells; cadherins may contribute to the sorting of heterogeneous cell types. KLHDC8B encodes a protein which forms a distinct beta-propeller protein structure of kelch domains (allowing for protein-protein interactions). Mutations have been associated with Hodgkin lymphoma.

Locus 3q21.2, rs142695226 (ITGB5 and UMPS): The product of ITGB5, integrin β5 is widely studied for it’s role in cell adhesion and integrin-mediated signaling. It plays a role in angiogenesis, overexpression promotes new blood vessel formation in vivo by enhancing the binding capacity of circulating angiogenic cells to endothelial cells, among other molecular effects [6]. UMPS encodes a uridine 5'-monophosphate synthase, it catalyzes the reaction of orotic acid and ribose-5-phosphate to uridine monophosphate (UMP), an energy-carrying molecule.

Locus 3q25.2, rs433903 (ARHGEF26(SGEF) and DHX36): ARHGEF26 (Rho Guanine Nucleotide Exchange Factor 26) encodes a member of the Rho-guanine nucleotide exchange factor (Rho-GEF) family. These proteins regulate Rho GTPases by catalyzing the exchange of GDP for GTP; ARGHEF26 is also named SGEF, reported to play a crucial role in atherosclerosis and is suggested to be a potential therapeutic target[7]. DHX36 is a gene which is a member of the DEAH-box family of RNA-dependent NTPases. It may be involved in regulation of telomere

length, function in sex development and spermatogenesis and may play a role in ossification [genecards].

Locus 4q21.21, rs10857147 (PRDM8 and FGF5): PRDM8 encodes a protein that belongs to a conserved family of histone methyltransferases that acts predominantly as negative regulators of transcription [genecards]. FGF5 is a member of the fibroblast growth factor family that play an important role in cell proliferation and differentiation; FGF5's major role is in regulation of hair length[8].

Locus 4q27, rs11723436 (MAD2L1 and PDE5A): MAD2L1 is a component of the mitotic spindle assembly checkpoint it prevents the anaphase, until all chromosomes at the metaphase are aligned. PDE5A (Phosphodiesterase 5A) is a Protein Coding gene. It is involved in the regulation of intracellular concentrations of cyclic nucleotides and is important for smooth muscle relaxation in the cardiovascular system. PDE5 expression is increased in patients with advanced cardiomyopathy[9].

Locus 4q31.22, rs35879803 (ZNF827): ZNF827 is a largely unknown zinc finger protein. It has been reported to recruit the NuRD (Nucleosome Remodeling Deacetylase) complex that has chromatin remodeling and histone deacetylase activities. The NuRD-ZNF827 complex promotes telomere-telomere recombination, it integrates and controls multiple mechanistic elements of 'alternative lengthening of telomeres' (ALT) activity [10].

Locus 6p22.3, rs35541991 (HDGFL1): HDGFL1 encodes hepatoma-derived growth factor-like 1. Variants near HDGFL1 have been genome wide associated with Total iron binding capacity[11] but its function remains to be determined.

Locus 11p15.2, rs1351525 (ARNTL): ARNTL (Aryl Hydrocarbon Receptor Nuclear Translocator Like), a transcriptional activator, and its product BMAL1, form the core components of the circadian clock and mainly known for interactions with CLOCK genes.

Locus 12q13.13, rs11170820 (HOXC4): There is not much known about HOXC4. HOXC4, is one of several HOXC genes located in a cluster on chromosome 12; three genes, HOXC5, HOXC4 and HOXC6, share a 5' non-coding exon. The homeobox genes encode a highly conserved family of transcription factors that play an important role in morphogenesis in all multicellular organisms. [genecards]. HOXC4 has been studied in relationship to differentiation of hematopoietic stem cells[12] and adipose tissue[13].

Locus 12q24.31, rs2244608 (HNF1A, OASL): HNF1A is a frequent cause of monogenic diabetes (MODY-HNF1A) and highly expressed in liver, pancreas and the proximal tubule of the kidney. It has been shown to be highly associated with lipid levels[14], and suggested to be involved in CRP, GGT, and other atherosclerotic and metabolic risk factors[15]. It plays a major role in the expression of various hepatic, renal, and pancreatic genes/proteins including megalin (Low density

lipoprotein-related protein 2), cubilin [16], PCSK9[17]. Altogether, HNF1A is a pleiotropic gene that is widely studied with many functions. OASL encods oligoadenylate synthetase enzymes, which are cytoplasmic dsRNA sensors belonging to the antiviral innate immune system.

Locus 14q24.3, rs3832966 (TMED10, NEK9, ZC2HC1C, RPS6KL1, EIF2B2 and ACYP1):

Little is known about TMED10's function. TMED10 is thought to be a type I membrane protein that is localized to the plasma membrane and golgi cisternae, involved in vesicular protein trafficking. NEK9 recently reported to be a cause for a lethal skeletal dysplasia. Loss of function results in defects of fibroblasts including a reduced proliferation capability and delayed cell cycle progression through the G1/S boundary and S-phase and could also be involved in ciliopathy[18]. EIF2B2 (Eukaryotic Translation Initiation Factor 2B Subunit Beta) is a Protein Coding gene. Diseases associated with EIF2B2 include Leukoencephalopathy With Vanishing White Matter and Late Infantile Cach Syndrome. Among its related pathways are Gene Expression and Translation Insulin regulation of translation [genecards]. ACYP1 is a member of the acylphosphatase family. The encoded protein is a small cytosolic enzyme that catalyzes the hydrolysis of the carboxyl-phosphate bond of acylphosphates. Two isoenzymes have been isolated and described based on their tissue localization: erythrocyte (common) type acylphosphatase encoded by this gene, and muscle type acylphosphatase [genecards]. nothing is known about the function of ZC2HC1C (Zinc Finger C2HC-Type Containing 1C) and RPS6KL1 (Ribosomal Protein S6 Kinase Like 1).

Locus 16q23.1, rs33928862 (BCAR1): Breast cancer anti-estrogen resistance protein 1 is a protein that in humans is encoded by the BCAR1 gene and involved in various cellular events, basic signaling of developmental/physiological processes and involved in regulation homeostasis of various tissues, BCAR1's functions and role has been reviewed previously[19]. A variant in LD (r^2=0.65), rs4888378, has been associated with Carotid Intima-Media Thickness and coronary artery disease risk[20] .

Locus 16q23.3 rs7500448 (CDH13): CDH13 is a widely studied member of de cadherin family, it is an adhesion glycoprotein known as T-cadherin and is recognized as an LDL receptor, although different to other LDL recepters, it activates Erk 1/2 tyrosine kinase and the nuclear translocation of NF-kappaB[21,22]. GVs near this gene have previously been genome wide associated with blood pressure[23] and adiponectin levels[24] ($P=6.8\times10^{-165}$), among others. The locus has also been identified in one of the first genome wide association studies of coronary artery disease[25], although not at genome wide significance. None of the reported SNPs were in LD (r^2>0.001) with the current finding, rs7500448. We identified rs7500448 to be highly associated ($P=8\times10^{-13}$) with pulse pressure in UK Biobank.

Locus 19q13.2, rs138120077 and rs8108632 (B9D2, TGFB1, HNRNPUL1 and CCDC97): not much is known about B9D2's function, the encoded protein localizes to basal bodies and cilia, mutations cause Meckel syndrome [26]. TGFB1, transforming growth factor beta1, is one of the most widely studied genes. It is a multifunctional peptide which regulates proliferation, differentiation, adhesion, migration, among other functions and studied for its role in angiogenesis, cardiovascular syndromes and vascular biology [27–29]. rs2241718 near TGBF1 has been prioritized as a functional regulatory variant[30] but is in low LD with the 2 signals identified in our study. The heterogeneous nuclear ribonucleoprotein U-like 1 (HNRPUL1) gene encoding for a hetero-geneous ribonuclear protein believed to be involved in mRNA processing and transport [31,32], candidate studies found significant associations between variants and CAD in high risk people with familial hypercholesteromelia [33]. Nothing is known for CCDC97 (Coiled-Coil Domain Containing 97), but it has been recently studied as a candidate for regulatory mechanisms of CAD, together with TGFB1[30]. This study showed that while the 3′-untranslated region variant at CCDC97/TGFB1, rs2241718, was predicted to affect binding, this variant might not alter endogenous CCDC97 levels, but rather serve as an enhancer for neighboring TGFB1 in human coronary artery smooth muscle cells.

Definitions used for UK Biobank analyses

Prevalent and incident coronary artery disease (CAD), hypercholesterolemia, hypertension, diabetes, myocardial infarction (MI), heart failure, atrial fibrillation / flutter, cerebral infarction was derived from self-reported (touchscreen questionnaire and verbal interview) and/or the diagnosis was captured using the Hospital Episode Statistics (HES) records (using the following ICD codes: I21 - I25 for CAD; E78 for hypercholesterolemia; I10 - I15 for hypertension; E10 - E14 for diabetes; I21 - I22 for MI; I42, I150 for heart failure; I48 for atrial fibrillation and flutter; I63 - I64 for cerebral infraction and transient ischemic attack; all codes beginning with I as primary diagnosis were used to define cardiovascular mortality. In addition information for CAD and device implantation (pacemaker or implantable cardioverter defibrillator) was captured through HES records which are coded according to the Office of Population Censuses and Surveys Classification of Interventions and Procedures, version 4 (OPCS-4) (using the following OPCS-4 codes: K40 - K46, K49, K50, K75 for CAD and; K59 - K61 and U31 for device implantation. The "Spell and Episode" category contains data relating to diagnoses made during hospital in-patient stay. It includes main and secondary diagnoses, coded according to the International Classification of Diseases (ICD). The main diagnosis corresponds to be the main reason for the hospital admission, while secondary diagnoses are more often contributory or underlying conditions. We used both the main and secondary diagnoses for recording prevalent and incident risk factors, conditions and events. For defining the control group we excluded participants who reported that their mother, father or sibling suffered form 'heart disease' (Field ID 20107, 20110 and 20111). Information on smoking status was collected using the touchscreen questionnaire at baseline visit. Medication usage was collected at the baseline visit during a verbal interview by a trained nurse on prescription medications (Field ID 20003). Data on beta block-blocker and calcium channel-blocker therapy was defined with corresponding medication codes (beta-blockers and calcium channel blockers, please see below for the exact codes that were used). Body mass index was calculated using BMI value constructed from height and weight measured during the initial Assessment Centre visit (Field ID 21001) and Body composition

estimation by impedance measurement (Field ID 23104). Blood pressure was measured using the manual reading (Field ID 93, 94) and automated reading (Field ID 4079, 4080) measurements. Pulse pressure was calculated by subtracting the diastolic from the systolic blood pressure value. Mean arterial pressure (MAP) was calculated by MAP = [(2 x diastolic blood pressure) + systolic blood pressure] divided by 3. When multiple measurements during firs visit were available the mean of all measurements were averaged and used in the analysis. In the UK Biobank cohort PWV for ASI assessment was measured using the PulseTrace PCA2 (CareFusion, San Diego, USA) (Field-ID 21021). The PulseTrace PCA2 uses finger photoplethysmography to obtain the pulse waveform during a 10-15 seconds measurement using an infrared sensor clipped to the end of the index finger[34]. When multiple measurements were available the mean of all measurements were averaged and used in the analysis.

Beta-blocker medication codes: 1140866724, 1140866738, 1140860192, 1140860292, 1140860404, 1140860308, 1140860312, 1141194804, 1140860316, 1140860320, 1140860322, 1140860332, 1140860336, 1140860340, 1141194808, 1140860342, 1140860418, 1140860422, 1140860426, 1140864950, 1140909368, 1141164276, 1141162898, 1141169516, 1141184722, 1140879758, 1140879760, 1140879762, 1140879818, 1140879822, 1140879824, 1140879830, 1140879834, 1140879842, 1140879854, 1140879866, 1141180778, 1141146124, 1141146126, 1141194810, 1141146128, 1140866692, 1140916342, 1140866704, 1140866764, 1140866766, 1140851556, 1140866778, 1140866782, 1140866784, 1140866798, 1140866802, 1140866800, 1140866804, 1140916730, 1140916868, 1140917076, 1141152076, 1140866712, 1141156754, 1141156808, 1141172742, 1140866726, 1140866756, 1140860172, 1140864410, 1140922930, 1140860232, 1140860244, 1140860250, 1140860266, 1140860274, 1140860278, 1140860180, 1140860194, 1140860212, 1140851576, 1140851480, 1140860220, 1140860222, 1140851484, 1140860230, 1140910614, 1140860294, 1140851492, 1140860304, 1140860362, 1140860380, 1140860382, 1140860386, 1140860390, 1140860394, 1140860396, 1140860398, 1140860400, 1140860402, 1140860406, 1140860410, 1140860314, 1140860318, 1140851508, 1140860324, 1140860328, 1140860330, 1140860334, 1140860338, 1140916628, 1140860348, 1141146184, 1140860352, 1140860356, 1140860358, 1140860434, 1140860492, 1141171152, 1141184324, 1141182904,

1141187780, 1140851522, 1140863724, 1140860498, 1141168498, 1141164280, 1141187048

Calcium-channel-blocker medication codes: 1141165470, 1141150926, 1141153328, 1140926778, 1140851784, 1140861088, 1140861114, 1140911088, 1141150538, 1141157140, 1141169730, 1140861190, 1140879802, 1140888646, 1140861276, 1140928226, 1141153394, 1140872568, 1140879806, 1140879810, 1140888510, 1141153026, 1140861128, 1140851730, 1140861130, 1140861136, 1140861138, 1140861166, 1140926780, 1141157136, 1140911698, 1141151474, 1140917428, 1140917452, 1141153454, 1140923618, 1140861176, 1140861090, 1140923572, 1140851794, 1140926188, 1140926966, 1140861110, 1140927934, 1140927940, 1140861120, 1141145870, 1141150500, 1140916930, 1141152600, 1141166752, 1141162546, 1140851798, 1140851800, 1140861194, 1140861202, 1141200400, 1140928212, 1141187094, 1141188152, 1141188576, 1141188836, 1141188920, 1141190160, 1141199858, 1141200782, 1141201814, 1140861282, 1140928234, 1141153032, 1141153400, 1141167832, 1141175224, 1141171804, 1141174684, 1141180238, 1141173766, 1141187962, 1141188936, 1141190548

Supplementary Figure 1 | Regional plots of the 15 novel genome wide associated loci with CAD. LD (R^2) was based on the Europeans of 1000 Genomes Phase 1 v3. P-values were based on the CARDIoGRAMplusC4D GWAS data to provide an accurate overview of the P-value distribution among variants at each locus.

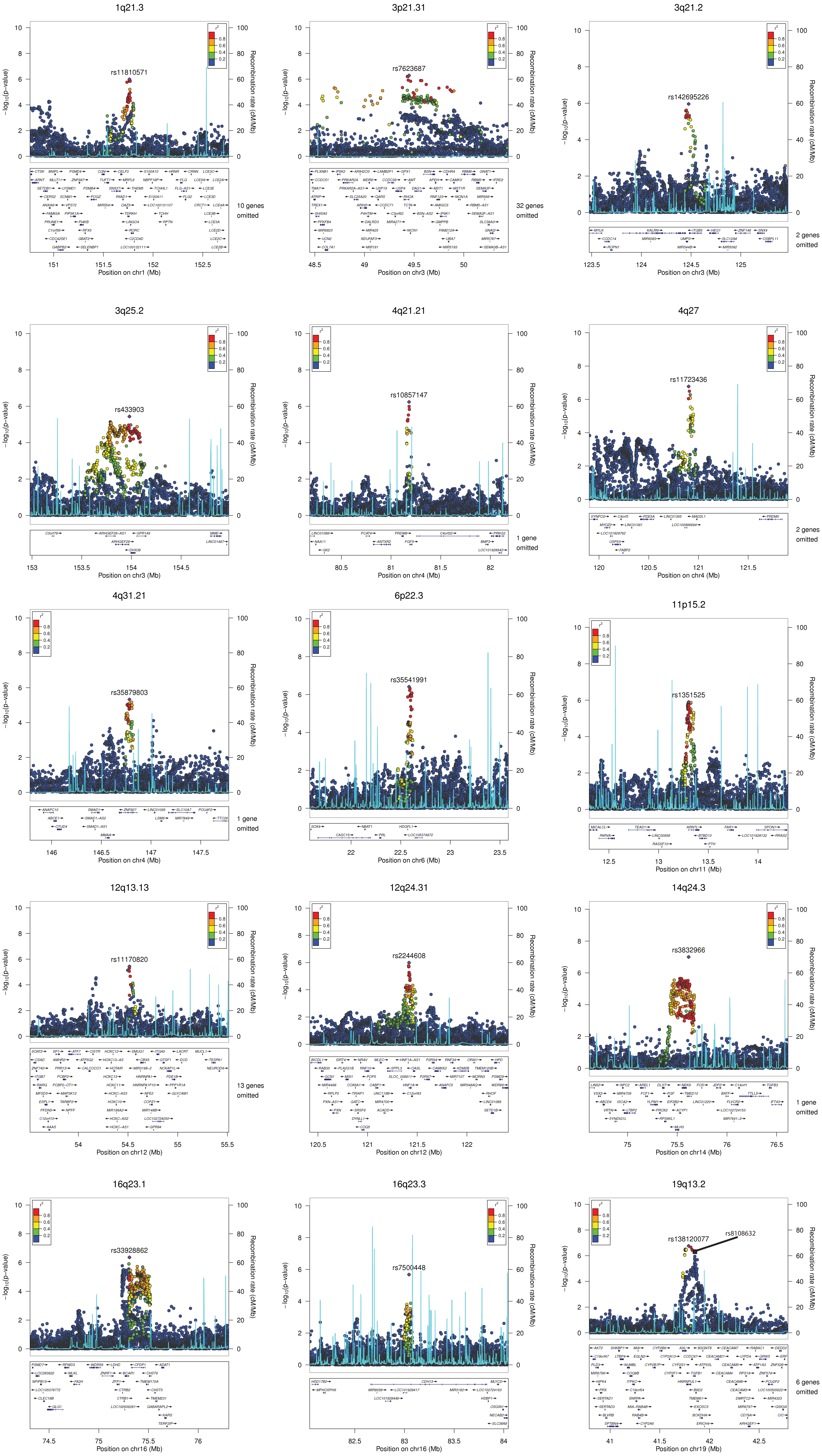

Supplementary References

1. Cook, D. N., Kang, H. S. & Jetten, A. M. Retinoic Acid-Related Orphan Receptors (RORs): Regulatory Functions in Immunity, Development, Circadian Rhythm, and Metabolism. *Nucl. Recept. Res.* **2,** (2015).
2. Zhang, Q. *et al.* Atorvastatin treatment improves the effects of mesenchymal stem cell transplantation on acute myocardial infarction: the role of the RhoA/ROCK/ERK pathway. *Int. J. Cardiol.* **176,** 670–679 (2014).
3. Van Hove, J., Coughlin, C. & Scharer, G. in *GeneReviews(®)* (eds. Pagon, R. A. et al.) (University of Washington, Seattle, 1993).
4. Kotake, S., Yago, T., Kawamoto, M. & Nanke, Y. The role of T-cell leukemia translocation-associated gene protein in human tumorigenesis and osteoclastogenesis. *J. Biomed. Biotechnol.* **2012,** 675317 (2012).
5. Nanke, Y. *et al.* A novel peptide from TCTA protein inhibits proliferation of fibroblast-like synoviocytes of rheumatoid arthritis patients. *Cent.-Eur. J. Immunol.* **39,** 468–470 (2014).
6. Leifheit-Nestler, M. *et al.* Overexpression of Integrin β5 Enhances the Paracrine Properties of Circulating Angiogenic Cells via Src Kinase–Mediated Activation of STAT3. *Arterioscler. Thromb. Vasc. Biol.* **30,** 1398–1406 (2010).
7. Samson, T. *et al.* The Guanine-Nucleotide Exchange Factor SGEF Plays a Crucial Role in the Formation of Atherosclerosis. *PLoS ONE* **8,** (2013).
8. Higgins, C. A. *et al.* FGF5 is a crucial regulator of hair length in humans. *Proc. Natl. Acad. Sci. U. S. A.* **111,** 10648–10653 (2014).

9. Pokreisz, P. *et al.* Ventricular phosphodiesterase-5 expression is increased in patients with advanced heart failure and contributes to adverse ventricular remodeling after myocardial infarction in mice. *Circulation* **119,** 408–416 (2009).

10. Conomos, D., Reddel, R. R. & Pickett, H. A. NuRD–ZNF827 recruitment to telomeres creates a molecular scaffold for homologous recombination. *Nat. Struct. Mol. Biol.* **21,** 760–770 (2014).

11. Li, J. *et al.* Genome-wide admixture and association study of serum iron, ferritin, transferrin saturation and total iron binding capacity in African Americans. *Hum. Mol. Genet.* **24,** 572–581 (2015).

12. Xin, C., Zhao, C., Yin, X., Wu, S. & Su, Z. Bioinformatics analysis of molecular mechanism of the expansion of hematopoietic stem cell transduced by HOXB4/HOXC4. *Hematol. Amst. Neth.* **21,** 462–469 (2016).

13. Singh, S., Rajput, Y. S., Barui, A. K., Sharma, R. & Datta, T. K. Fat accumulation in differentiated brown adipocytes is linked with expression of Hox genes. *Gene Expr. Patterns* **20,** 99–105 (2016).

14. Global Lipids Genetics Consortium. Discovery and refinement of loci associated with lipid levels. *Nat. Genet.* **45,** 1274–1283 (2013).

15. Reiner, A. P. *et al.* Common coding variants of the HNF1A gene are associated with multiple cardiovascular risk phenotypes in community-based samples of younger and older European-American adults: the Coronary Artery Risk Development in Young Adults study and the Cardiovascular Health Study. *Circ. Cardiovasc. Genet.* **2,** 244–254 (2009).

16. Terryn, S. *et al.* Tubular proteinuria in patients with HNF1α mutations: HNF1α drives endocytosis in the proximal tubule. *Kidney Int.* **89,** 1075–1089 (2016).

17. Shende, V. R. *et al.* Reduction of circulating PCSK9 and LDL-C levels by liver-specific knockdown of HNF1α in normolipidemic mice. *J. Lipid Res.* **56,** 801–809 (2015).

18. Casey, J. P. *et al.* Recessive NEK9 mutation causes a lethal skeletal dysplasia with evidence of cell cycle and ciliary defects. *Hum. Mol. Genet.* ddw054 (2016). doi:10.1093/hmg/ddw054

19. Camacho Leal, M. del P. *et al.* p130Cas/BCAR1 scaffold protein in tissue homeostasis and pathogenesis. *Gene* **562,** 1–7 (2015).

20. Boardman-Pretty, F. *et al.* Functional Analysis of a Carotid Intima-Media Thickness Locus Implicates BCAR1 and Suggests a Causal Variant. *Circ. Cardiovasc. Genet.* **8,** 696–706 (2015).

21. Rubina, K. A., Kalinina, N. I., Parfyonova, Y. V. & Tkachuk, V. A. T-cadherin as a receptor regulating angiogenesis and blood vessel remodeling. *Biochem. Mosc. Suppl. Ser. Membr. Cell Biol.* **1,** 57–63 (2007).

22. Kipmen-Korgun, D. *et al.* T-cadherin mediates low-density lipoprotein-initiated cell proliferation via the Ca(2+)-tyrosine kinase-Erk1/2 pathway. *J. Cardiovasc. Pharmacol.* **45,** 418–430 (2005).

23. Org, E. *et al.* Genome-wide scan identifies CDH13 as a novel susceptibility locus contributing to blood pressure determination in two European populations. *Hum. Mol. Genet.* **18,** 2288–2296 (2009).

24. Wu, Y. *et al.* A meta-analysis of genome-wide association studies for adiponectin levels in East Asians identifies a novel locus near WDR11-FGFR2. *Hum. Mol. Genet.* **23,** 1108–1119 (2014).

25. Wellcome Trust Case Control Consortium. Genome-wide association study of 14,000 cases of seven common diseases and 3,000 shared controls. *Nature* **447,** 661–678 (2007).

26. Dowdle, W. E. *et al.* Disruption of a ciliary B9 protein complex causes Meckel syndrome. *Am. J. Hum. Genet.* **89,** 94–110 (2011).

27. ten Dijke, P. & Arthur, H. M. Extracellular control of TGFβ signalling in vascular development and disease. *Nat. Rev. Mol. Cell Biol.* **8,** 857–869 (2007).

28. Goumans, M.-J., Liu, Z. & ten Dijke, P. TGF-β signaling in vascular biology and dysfunction. *Cell Res.* **19,** 116–127 (2009).

29. Pardali, E., Goumans, M.-J. & Dijke, P. ten. Signaling by members of the TGF-β family in vascular morphogenesis and disease. *Trends Cell Biol.* **20,** 556–567 (2010).

30. Miller, C. L. *et al.* Integrative functional genomics identifies regulatory mechanisms at coronary artery disease loci. *Nat. Commun.* **7,** 12092 (2016).

31. Gabler, S. *et al.* E1B 55-Kilodalton-Associated Protein: a Cellular Protein with RNA-Binding Activity Implicated in Nucleocytoplasmic Transport of Adenovirus and Cellular mRNAs. *J. Virol.* **72,** 7960–7971 (1998).

32. Kzhyshkowska, J., Rusch, A., Wolf, H. & Dobner, T. Regulation of transcription by the heterogeneous nuclear ribonucleoprotein E1B-AP5 is mediated by complex formation with the novel bromodomain-containing protein BRD7. *Biochem. J.* **371,** 385–393 (2003).

33. van der Net, J. B. *et al.* Replication study of 10 genetic polymorphisms associated with coronary heart disease in a specific high-risk population with familial hypercholesterolemia. *Eur. Heart J.* **29,** 2195–2201 (2008).

34. UK Biobank. *UK Biobank Arterial Pulse-Wave Velocity*
